# #2714, a novel active inhibitor with potent G2/M phase arrest and antitumor efficacy in preclinical models

**DOI:** 10.1038/s41420-018-0032-y

**Published:** 2018-02-14

**Authors:** Wen-Jie Lu, Wen Peng, Qian-Qian Sun, Yong-Huai Li, Bo Chen, Luo-Ting Yu, You-Zhi Xu, Si-Ying Wang, Ying-Lan Zhao

**Affiliations:** 1 School of Basic Medicine, Anhui Medical University, Hefei, 230032 China; 2 Department of Oncology, The People’s Hospital of Guizhou Province, Guiyang, 550004 China; 3 State Key Laboratory of Biotherpay, Cancer Center, West China Hospital, Sichuan University, Chengdu, 610041 China; 4 Department of Respiratory Disease, The First Affiliated Hospital of Anhui Medical University, Hefei, Anhui 230022 China; 5 Department of Gastrointestinal Surgery, The First Affiliated Hospital of Anhui Medical University, Hefei, Anhui 230022 China

## Abstract

Arresting cell cycle has been one of the most common approaches worldwide in cancer therapy. Specifically, arresting cells in the G2/M phase is a promising therapeutic approach in the battle against lung cancer. In the present study, we demonstrated the anticancer activities and possible mechanism of compound #2714, which can prompt G2/M phase arrest followed by cell apoptosis induction in Lewis lung carcinoma LL/2 cells. In vitro, #2714 significantly inhibited LL/2 cell viability in a concentration- and time-dependent manner while exhibiting few toxicities on non-cancer cells. The mechanism study showed that cell proliferation inhibition due to the treatment with #2714 correlated with G2/M phase arrest and was followed by LL/2 cell apoptosis. The characterized changes were associated with the downregulation of phosphorylated cell division cycle 25C (Cdc25C) and upregulation of p53. Apoptosis-associated activation of cleaved caspase-3 was also detected. Moreover, #2714 strongly attenuated LL/2 cell proliferation by disrupting the phosphorylation of p44/42 mitogen-activated protein kinase (MAPK). In vivo, intraperitoneal administration of #2714 (25–100 mg/kg/day) to mice bearing established tumors in xenograft models significantly prevented LL/2 tumor growth (58.1%) without detectable toxicity. Compound #2714 significantly increased apoptosis in LL/2 lung cancer cells in mice models, as observed via terminal deoxynucleotidyl transferase (TdT) dUTP nick-end labeling (TUNEL) assay, and the data from an immunohistochemical analysis showed that #2714 remarkably inhibited the proliferation and angiogenesis of lung cancer in vivo. Taken together, our data suggest that #2714 has a high potential anti-lung cancer efficacy with a pathway-specific mechanism of G2/M phase arrest and subsequent apoptosis induction both in vitro and in vivo*;* its potential to be an anticancer candidate warrants further investigation.

## Introduction

Lung cancer has become a common and major public health problem worldwide^1,2^. Lung cancers have been ranked as number one in causes of cancer-related disease and deaths^3,4^. However, the survival status of lung cancer is very poor;^5,6^ the dismal 5-year survival rate has risen just a small percentage over the several past decades and current lung cancer therapy is far from adequate^7–9^. Therefore, there is an urgent need to develop novel drugs that show stronger efficacy along with robust safety profiles in order to conquer this malignancy.

**Fig. 1:**
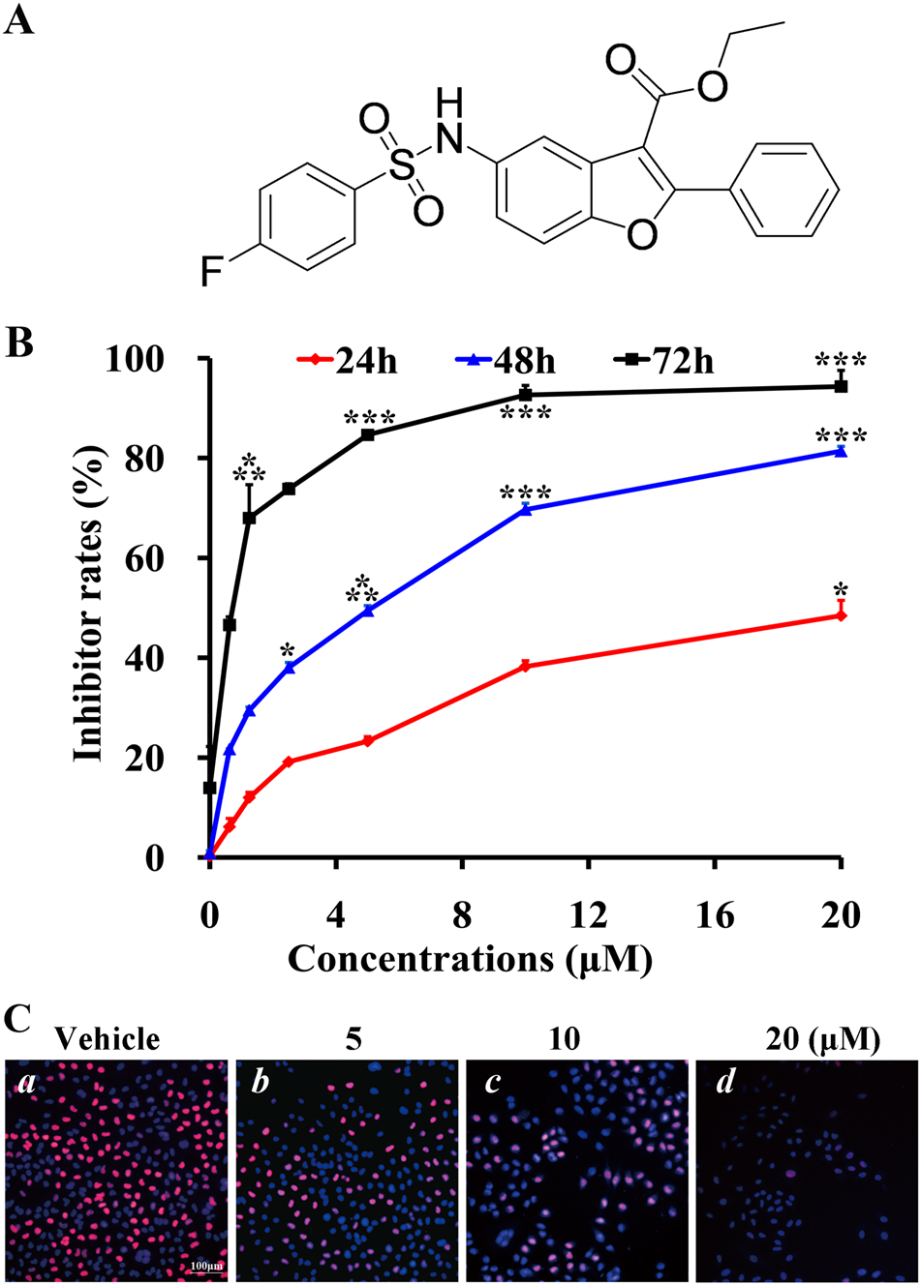
#2714 blocked cellular proliferation and induced apoptosis in vitro. **a** The chemical structure of compound #2714. **b** The CCK-8 assay was used to measure the inhibition effects of #2714 on Lewis lung carcinoma (LL/2) cells after treatment for different times and concentrations. **c** The EdU–DNA incorporation assay was carried out to evaluate the effect of #2714 on LL/2 cell proliferation. The fluorescence microscopic appearance of S-phase LL/2 cells were stained with Hoechst 333258 (blue) and EdU (red) after treatment with different concentrations of #2714 for 48 h. (Mean ± SD, *n* = 3; 100×; *p < 0.05; ***p < 0.001)

The use of chemosynthetic small molecular targeted drugs has shown to be effective in treating lung cancer^10–12^. Over the past few years, we have been trying to find new candidates for lung cancer treatment and have reported several small molecule candidates for lung cancer therapy via computer-aided drug design (CADD) and high-throughput screening approaches. Using our previous observations, we modified the structure of compound YL4073, which had shown profound effects against lung cancer^12^, and obtained a novel small molecule compound named as #2714 (Fig. 1a). To the best of our knowledge, this new compound has shown some profound pharmacological properties including blocking the proliferation of lung cancer cells, and it is possible that #2714 might have potential pharmacodynamic applications as a medicinal product using its novelty ability to arrest the cell cycles of other lung cancer cells, as well.

Cell cycle regulation plays a key role in cell proliferation and in the survival process of lung cancer. The progression of every phase of the cell cycle can be regulated by cell cycle regulators in cell cycle-associated signal pathways. For example, the enzyme polo-like kinase 1 (Plk1) plays an important part in the G2/M phase transition procession, whereas the levels and/or activity of phosphorylated cell division cycle 25C (Cdc25C) helps maintain the G2 phase^13–15^. The protein levels and/or activity of phosphorylated p44/42 mitogen-activated protein kinase (MAPK) and phosphorylated Akt also play an important role in the G2 phase^16,17^. Indeed, many researchers have reported the abnormal expression of Plk1 and Cdc25C in several cancer cells. In particular, it is noteworthy that the loss control of the G2/M phase transition plays an important role in lung cancer proliferation and apoptosis^18^. Therefore, overcoming malignant cell proliferation and cancer cell survival via cell cycle arrest is a promising strategy for developing novel small molecule targeted agents.

The current study was designed to elaborate on the activities and possible molecular mechanisms of the potent agent #2714, which caused murine Lewis lung carcinoma LL/2 cell death and G2/M phase cell cycle arrest.

We focused mainly on lung carcinoma due to the poor prognosis and lack of effective therapies in treating this tumor type. We have shown that #2714 is a potent small molecular agent that results in G2/M phase arrest in LL/2 cells. Cell apoptosis was followed by G2/M phase cell cycle arrest and involved caspase-dependent apoptosis and MAPK signaling pathways. In addition, #2714 significantly inhibited the growth of an LL/2 tumor model in vivo. Our results indicate that #2714 has high anti-lung cancer efficacy and that G2/M phase arrest is a pathway-specific mechanism of #2714.

## Results

### Effect of #2714 on cell viability in vitro

To measure the effect of #2714 on cell viability, a series of cancer cell lines was exposed to #2714 for 48 h and cell viability was detected via CCK-8 assay. Table 1 shows that #2714 significantly inhibited cancer cell viability. The IC_50_ value was 6.12 μM for the mammary carcinoma cell line 4T1 and 5.43 μM for the Lewis lung carcinoma LL/2 cell line. Of note, #2714 had a remarkable safety profile on the normal cell line NIH-3T3 cell, with an IC_50_ value that was eightfold higher than that of the cancer cell lines. Moreover, the inhibition effects of #2714 on LL/2 cells were time and concentration dependent (Fig. 1b).

**Table 1:** The effects of compound #2714 on cancer and non-cancer cellular viability

Cell line	Cell line type	IC_50_ (μM)
LL/2	Murine lewis lung carcinoma cell	5.43
4T1	Mouse mammary carcinoma cell	6.12
NIH-3T3	Mouse fibroblasts cell	å 40
HK-2	Human proximal tubular cell	å 40

A CCK-8 assay was carried out to measure the effects of #2714 on cell viability. All cell lines were exposed to different concentrations (cancer lines 0–20 μM; non-cancer lines 0–40 μM) of #2714 for 48 h. Data were expressed as mean of IC_50_; *n* = 3

### In vitro effects of #2714 on LL/2 determined via DNA labeling with 5-ethynyl-2′-deoxyuridine (EdU)

The EdU–DNA incorporation assay was used to further evaluate the inhibitory effect of #2714 on LL/2 cell proliferation. Our results show that the percentage of S-phase cells was 56.0% following exposure to 5 μM #2714. Moreover, when cells were exposed to 10 μM and 20 μM #2714, the percentages of viable S-phase cells decreased to 36.7% and 6.6%, respectively (Fig. 1c). These data suggest that #2714 blocks LL/2 cellular proliferation in a concentration-dependent manner.

### In vitro effects of #2714 on inducing cells cycle arrest

**Fig. 2:**
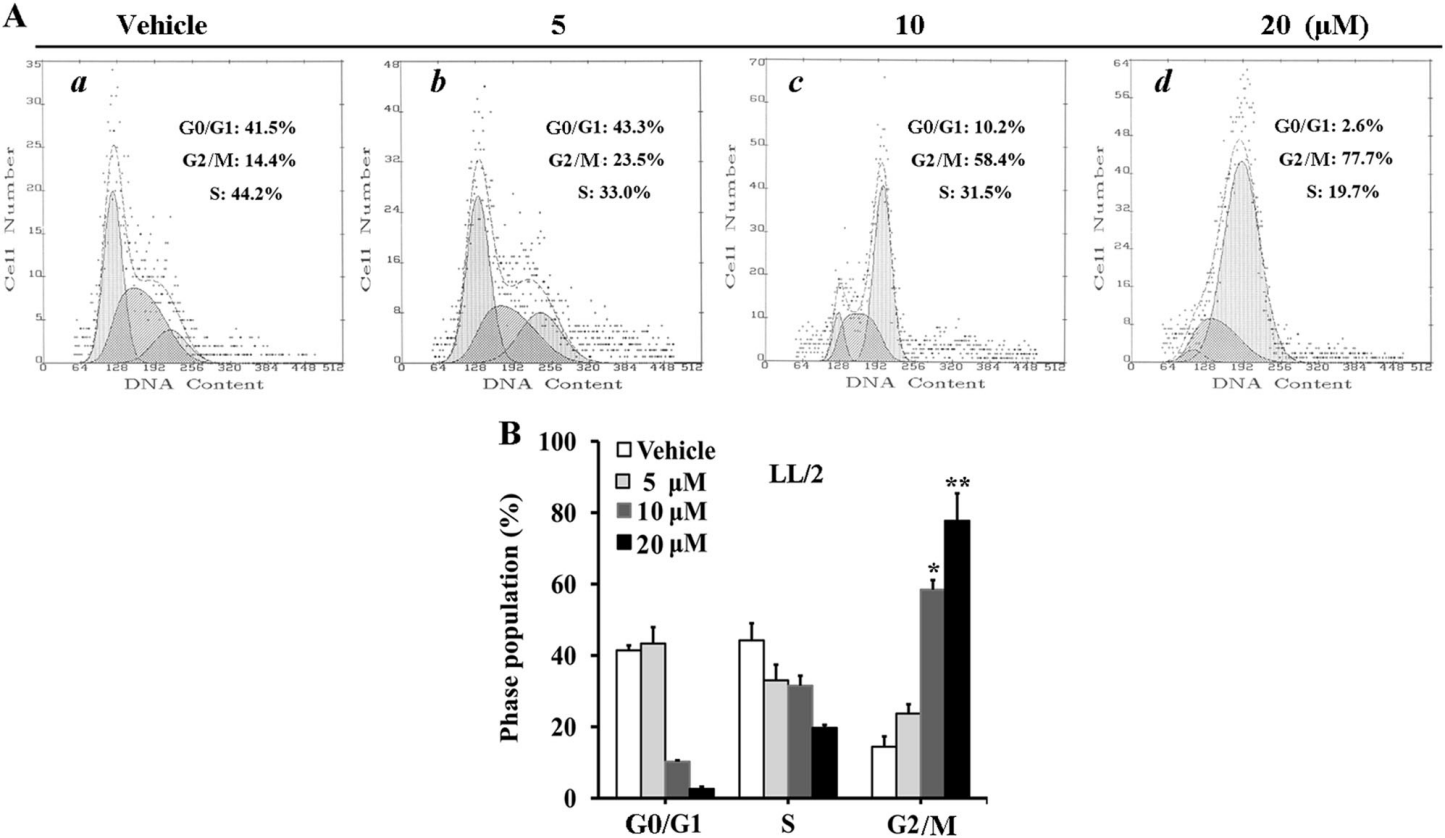
Effects of compound #2714 on the induction of G2/M phase arrest in Lewis lung carcinoma (LL/2) cells, as analyzed by flow cytometry in vitro. **a** Cells were treated with #2714 at the specified time, and the LL/2 cell cycles were analyzed. **b** The bottom panel shows the effect of #2714 on G0/G1, S, and G2/M phases compared with control. The histogram graphs and data shown are representative of three parallel and independent experiments (mean ± SD, *n* = 3; *p < 0.05; **p < 0.01)

We next analyzed cell cycle distribution via flow cytometry (FCM) to determine the possible mechanism responsible for #2714-mediated cell proliferation inhibition. Our data showed that 20 μM #2714 could suppress cell growth, and resulted in a remarkable accumulation of cells in G2/M accompanied by a decrease in S and G0/G1 DNA content (Fig. 2a). After the exposure of LL/2 cells to 20 μM #2714 for 48 h, the percentage of cells in G2/M phase increased to 77.7% (Fig. 2b).

### In vitro effects of #2714 on inducing cells apoptosis

**Fig. 3:**
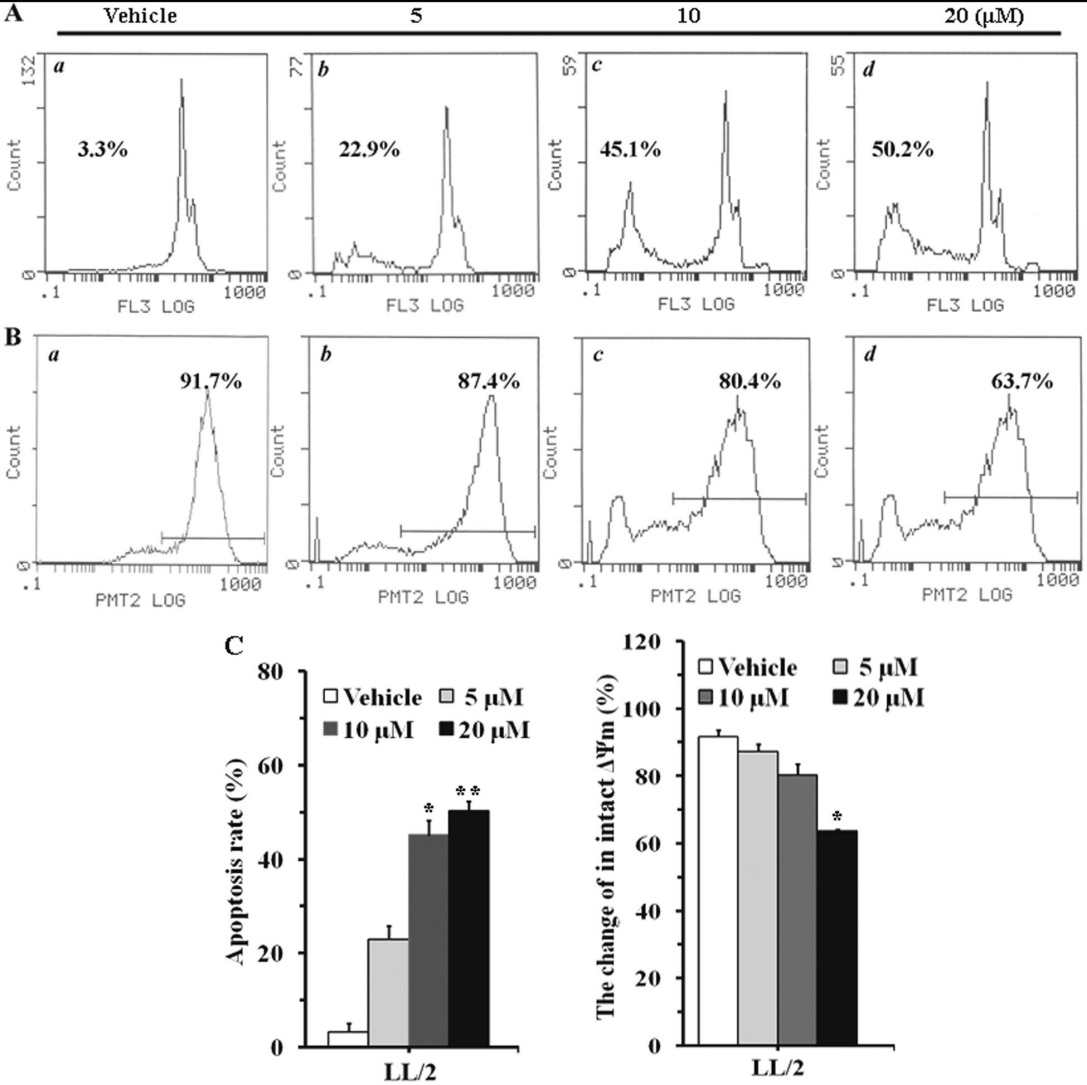
Effects of compound #2714 on inducing apoptosis in LL/2 cells, as analyzed by flow cytometry in vitro. **a** The morphological changes and apoptosis induction, concentration-dependent effects, were measured after Lewis lung carcinoma (LL/2) cells were treated with compound #2714 for 48 h and stained with propidium iodide (PI). **b** The effects of #2714 on mitochondrial membrane potential (ΔΨm) were detected using Rho 123 staining after LL/2 cells were treated with #2714 for 48 h. **c** The histogram graphs and data shown are representative of three parallel and independent experiments (mean ± SD, *n* = 3; *p < 0.05; **p < 0.01)

The morphological changes and apoptosis induction effect was measured after LL/2 cells were treated with #2714 for 48 h. Our data showed that the percentage of apoptosis cells in #2714-treated group increased in a concentration-dependent manner (Fig. 3). For example, the percentage of apoptosis cells was 22.9% in the 5 μM #2714-treated group, whereas the apoptosis rates increased to 45.1% and 50.2% when LL/2 cells were treated with 10 μM and 20 μM #2714, respectively, for 48 h (Fig. 3a).

Moreover, the effects of #2714 on ΔΨm were detected using Rho 123, because the mitochondrial membrane permeability disruption and loss of ΔΨm, which have been reported to play a key role in apoptosis are associated with a lack of Rho 123 retention fluorescence^12^. Our data showed that #2714 resulted in a significant decrease in Rho 123 fluorescence in intact LL/2 mitochondrial membranes after treatment for 48 h (Fig. 3b), suggesting that the collapse of ΔΨm in intact mitochondrial membranes was induced by #2714 and detected by FCM.

### #2714-induced G2/M arrest, which occurred through decreasing p-Cdc25C and upregulating p53 in LL/2 cells

**Fig. 4:**
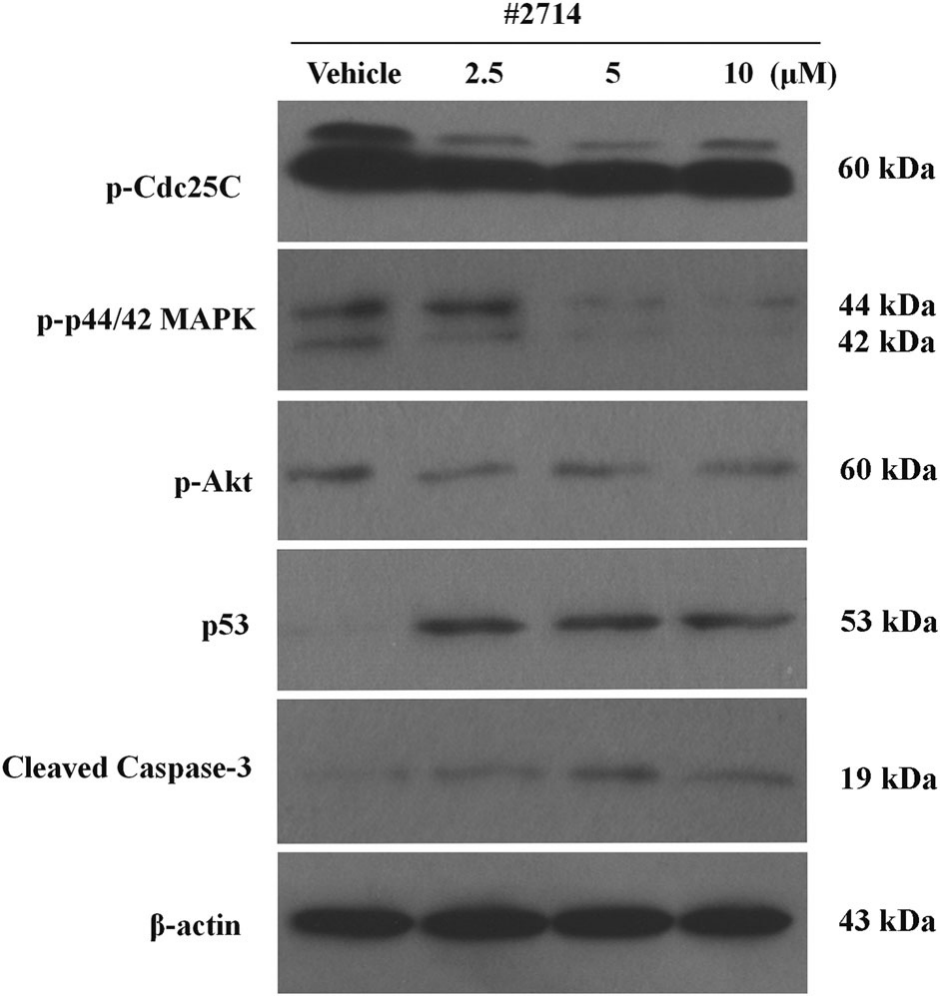
Effects of compound #2714 on the expression of key proteins involved in the G2/M phase transition and on apoptosis- and proliferation-associated proteins, analyzed via western blotting. Lewis lung carcinoma (LL/2) cells were exposed to compound #2714 for 24 h at designated gradient concentrations ranging from 2.5 to 10 μM in vitro. The expression levels of the phosphorylated forms of Cdc25C, Akt, and p44/42 MAPK in LL/2 cells were analyzed after treatment. The expression levels of p53 and cleaved caspase-3 were also assessed via western blotting. β-Actin was used as a loading control. Data are representative of three independent and parallel experiments

To investigate the possible mechanism of the G2/M arrest, we measured the expression levels and activations of p-Cdc25C after exposure to #2714, because Cdc25C plays an important role in regulating the G2/M cell cycle. As shown in Fig. 4, after treatment for 24 h, #2714 significantly inhibited the expression levels of p-Cdc25C in LL/2 cells. The expression levels of p53 were then analyzed to determine whether this protein plays a role in the regulation of G2/M arrest in LL/2 cells. Our data showed that #2714 increased the expression level of p53 after treatment for 24 h.

### #2714 activated cleaved caspase-3 and decreased phosphorylated (p-)p44/42 MAPK and p-Akt levels

The expression of caspase proteins was measured after the exposure to #2714 for 24 h in order to investigate whether #2714-induced apoptosis was associated with the caspase family. Our data showed that the expression of cleaved caspase-3 increased remarkably in a concentration-dependent manner (Fig. 4), suggesting that caspase-3 was activated by #2714. Moreover, we analyzed the expression level of p-p44/42 MAPK and p-Akt to determine whether exposure to #2714 caused inhibition of LL/2 proliferation. Our data showed that the expression of p-p44/42 MAPK and p-Akt were downregulated after #2714 treatment.

### Safety profile of #2714 in acute toxicity evaluation

**Fig. 5:**
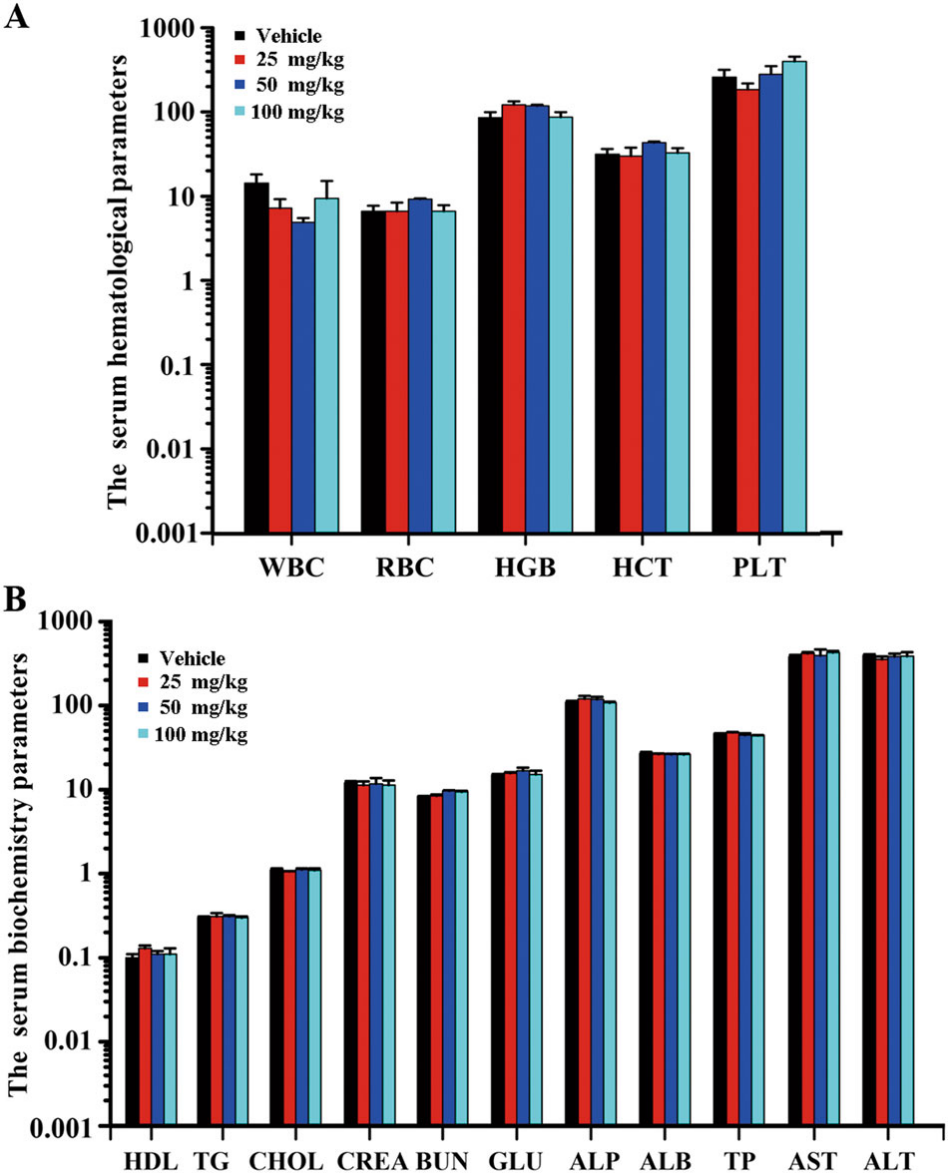
Evaluation of the safety profile for compound #2714 using an acute toxicity test in vivo. **a** Hematological and **b** serum biochemical values were determined from the blood samples collected from experimental animals killed at the end of the 14-day acute toxicity experiment. (Mean ± SEM, *n* = 10)

To evaluate the safety profile of #2714 in vivo, an acute toxicity test was carried out using C57BL/6 mice. Our data showed that #2714 was well tolerated during the 14-day experiment period; there were no obvious changes in mortality, and no adverse clinical signs or symptoms of toxicity were observed in any experimental mice. There were no significant differences in hematological and serum biochemical data (Figs. 5a, b), and no obvious pathological damage (data not shown) after the exposure to 25–100 mg/kg #2714 for 2 weeks. In addition, the no-observed-adverse-effect-level or #2714 was >1000 mg/kg.

### #2714 showed a good pharmacodynamic profile in vivo

**Fig. 6:**
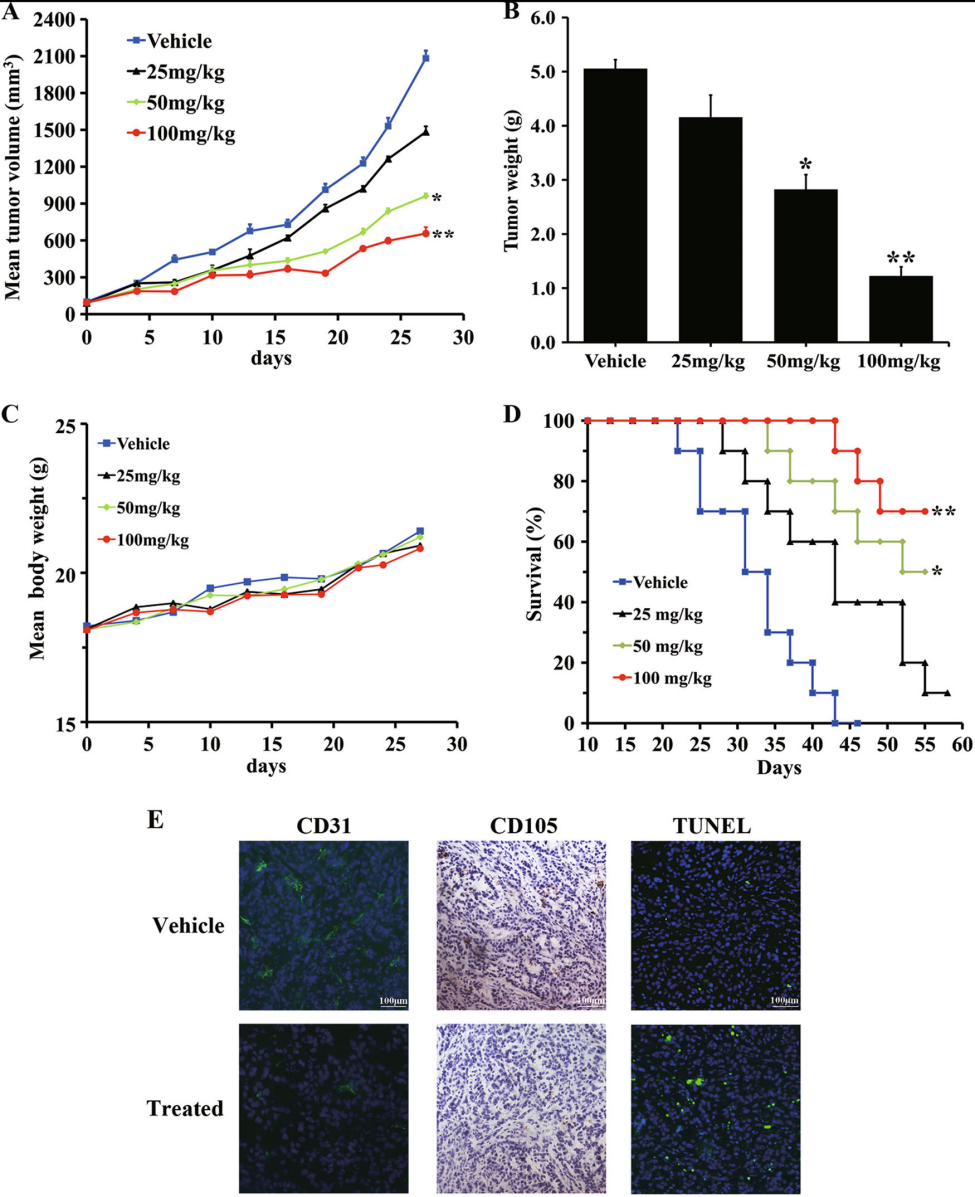
Compound #2714 showed a strong anticancer effect and pharmacodynamics profile in vivo. **a** Lewis lung carcinoma (LL/2) cells were injected subcutaneously into female C57BL/6 mice and inoculated for 10 days. These mice, with LL/2-bearing tumors, were then intraperitoneally (i.p.) administered with 100, 50, or 25 mg/kg #2714; vehicle was used as a control. Tumor volumes were measured every other day for 14 days starting from the initiation of treatment. **b** Growth inhibition rates were measured by weighing the tumors of experimental animals killed at the end of the experiment after #2714 was administered to LL/2-bearing mice xenograft models. **c** The average body weight changes of each group were measured every other day. **d** A significant increase in survival in #2714-treated mice was observed. **e** An immunohistochemical analysis was conducted to detect the effects of #2714 on tumor angiogenesis inhibition and apoptosis induction by detecting CD31 and CD105 after animals were administered #2714 for 14 days. A TUNEL assay was used to detect the LL/2-bearing tumor mice xenograft sections. The representative fields of tumor sections from the TUNEL assay were observed under an inverted fluorescence microscope with a camera (log-rank, mean ± SEM, *n* = 10, 200×; *p < 0.05, **p < 0.01)

To study the antitumor activity of #2714 in vivo, LL/2-bearing C57BL/6 mice were administered 100, 50, or 25 mg/kg #2714 by intraperitoneal (i.p.) injection, when the mice were inoculated with tumors that reached the size of approximately 70–100 mm^3^. Continuous tumor growth was observed and tumor volumes were measured every other day for 28 days from the initiation of treatment. At the end of the experiments, the LL/2-bearing mice were killed and the tumor weights were weighed using an electronic scale. As shown in Figs. 6a, b, there were decreases of 45.1% and 58.1% tumor growth after the mice were treated with 50 and 100 mg/kg #2714, respectively, compared with the control, suggesting that #2714 exhibited a strong antitumor activity by inhibiting tumor growth. Furthermore, there was no significant change in the body weight curve (Fig. 6c). These results indicate that #2714 had high efficacy and low toxicity. Moreover, as shown in Fig. 6d, a significant increase in survival in #2714-treated mice was observed (log-rank, mean ± SEM, *n* = 10, *p < 0.05, **p < 0.01).

### #2714-induced cell apoptosis

The immunohistochemical and terminal deoxynucleotidyl transferase (TdT) dUTP nick-end labeling (TUNEL) analyses were conducted to detect whether #2714 could inhibit tumor proliferation and angiogenesis, and induce tumor cell apoptosis in vivo. As shown in Fig. 6e and indicated by CD31 and CD105 staining, which showed a 37.4% and 15.2% decrease compared with control samples, respectively, #2714 significantly arrested the cell cycle and markedly decreased angiogenesis in LL/2 tumor tissues treated with #2714 for 14 days. Moreover, the TUNEL assay found that there were a dramatically higher number of apoptotic tumor cells after treatment with #2714 (11-fold increase vs. vehicle).

## Discussion

Considerable progress has been made in malignancy lung cancer therapy, but many serious challenges still remain^19,20^. Recently, research has indicated that chemotherapy causes poor effects on the survival rate of patients with advanced lung cancer, notwithstanding that it was used as the clinical first-line treatment strategies for patients with advanced lung cancers^20^. Therefore, it is fundamentally urgent to meet the current medical needs and find a more rational and effective treatment for block advanced lung cancers^21,22^. In this paper, we identified a candidate compound named as #2714, which could arrest the G2/M phase and induced apoptosis in murine Lewis lung cancer LL/2 cells both in vitro and in vivo.

#2714 was obtained through the use of CADD and structural modification targeted cell cycle kinases, the chemical structure of which is novel and is different from cell cycle kinase inhibitors approved for clinical use. In a previous cell-based screening assay, #2714 significantly inhibited the proliferation of a series of murine cancer cell lines, and LL/2 cells were found to be the most sensitive with an, IC_50_ value for LL/2 cells of 5.43 μM. Therefore, we choose LL/2 cells to investigate the cell cycle arrest and apoptosis associated with #2714. The anti-proliferation effect of #2714 was further measured using an EdU–DNA incorporation assay in LL/2 cells. Moreover, the safety profiles of #2714 showed a wide therapeutic window with high IC_50_ values in non-cancer cell line suggesting it has a selective cytotoxicity effect on cancer lines. Up to now, there were hardly any G2/M inhibitors been approved in clinical use because of their toxicities^23,24^.

Mechanistically, the effect of #2714 on cell cycle arrest may be the main mechanism that inhibits tumor growth. There is a tightly regulated system that controls the eukaryotic cell proliferation process. The major regulatory checkpoint is the transition from the G2 to the M phase, and the reaction of this transition is catalyzed by Plk1 and p-Cdc25C^22–24^. Our data show that #2714 potently downregulated the expression levels and activity of p-Cdc25C, resulting in G2/M cell cycle arrest and inhibition of cell proliferation that occurred in a concentration-dependent manner in LL/2 cells. Moreover, in G2/M phase regulation, p53 regulates the cell cycle process and mediates p53-dependent G2 arrest in the G2/M transition by blocking the levels and/or activities of Plk1 and p-Cdc25C; thus, p53 inhibits cell proliferation by inducing cancer cell cycle arrest^25^. In this study, our results showed that the expression of p53 in LL/2 cells increased after exposure to #2714. Therefore, #2714 may have stimulated the G2/M arrest that is mediated by p53. Although we have detected the potential and possible targets of #2714, the inhibition of p-Cdc25C still cannot clarify the specific activity of #2714 in LL/2 cells. Further studies using bioinformatics predictions are needed to investigate and confirm the detailed and possible specific sensitivity mechanism to #2714 treatments in LL/2 cells. Taken together, we speculate that #2714 might induce p-Cdc25C reduction, which finally results in G2/M cell cycle arrest. These results indicate that #2714 has the potential to be Cdc25C inhibitor candidate and might be developed as a new cell cycle inhibitor.

We also evaluated the in vivo anticancer effect of #2714 in an established LL/2 lung cancer model in C57BL/6 mice. These models have been applied for investigating the pharmacodynamics and mechanism of candidate agents in vivo. Our data showed that the effective dose of 100 mg/kg #2714 could block the growth of murine lung cancer cells by 58.1% in the C57BL/6 mouse model. Moreover, #2714 could reduce cancer angiogenesis and cell proliferation, in addition to its effects on arresting cell cycle and inducing apoptosis in vivo. In addition, the data from the acute toxicity test suggested that #2714 has a good safety profiles; for example, there was no obvious difference in the hematological and serum biochemical data between the test and control groups.

These results suggest #2714 targeted to specific targets of cancer. However, it’s still a problem to be solved that what is its primary target. We have tried some limited study study and new methods to identify #2714’s new potential target. For example, we used a chemical biology method named as IFPTarget, which uses an interaction fingerprinting (IFP) method based on protein–ligand IFP analyses for target-specific interaction analyses and a comprehensive index (Cvalue) for the target ranking test set by CADD^26–28^, to investigate and searching the possible new potential targets for #2714 and found that ProCaspase-6, soluble epoxide hydrolase, peroxisome proliferator-activated receptor γ, Ser/thr-Transferase B-raf (V600E), mitogen-activated transferase kinase kinase kinase 4 (MAP4K4), caspase-7, and cyclin-dependent kinase 2 (CDK2) might be the new potential targets (data not shown). We profiled enzymatic kinase inhibition activity and found that #2714 could inhibit the activities of caspase-6, caspase-7, and CDK2. Therefore, we speculate that cell cycle arrest- and apoptosis-related protein might be its new potential target from the results of the present study. Recently, years, quantitative chemical proteomics, and metabolomics in chemical biology have been demonstrated as the effective to identify the new potential targets of small molecules^29,30^. Further and in-depth study needs to be applied to investigate the target for #2714.

In summary, our study suggested that #2714 arrested cell cycle progression and induced apoptotic cell death, which contributed to its anti-proliferation and anti-angiogenesis effects against murine Lewis lung carcinoma LL/2 cells both in vitro and in vivo. The most probable mechanism underlying #2714-induced apoptosis is the activation of cleaved caspase-3, the upregulation of p53, and the downregulation of p-p44/42 MAPK and p-Akt, which together lead to the downregulation of G2/M phase-related cell cycle-dependent kinases such as p-Cdc25C and result in G2/M phase arrest and apoptosis in LL/2 cells. The potential of #2714 to be an anti-lung cancer candidate compound warrants further investigation and the inhibition of cyclin kinases is a promising approach to cancer therapy.

## Materials and methods

### Materials

Dimethyl sulfoxide (DMSO), propidium iodide (PI), and rhodamine 123 (Rho 123) were purchased from Sigma Chemical Co. (St. Louis, MO). Cell Counting Kit-8 (CCK-8) was purchased from Dojin do Laboratory (Dojin, Japan). The primary antibodies for p-Cdc25C, p53, p-p44/42 MAPK, and cleaved caspase-3 were purchased from Cell Signaling Technology (Beverly, MA). The primary antibodies for anti-CD31 and anti-CD105 were purchased from BD Biosciences Co. (Franklin Lakes, NJ, USA). β-Actin and the horseradish peroxidase-conjugated secondary antibodies were purchased from Santa Cruz Biotechnology (Santa Cruz, CA). The TUNEL assay kit was purchased from Promega (Madison, WI, USA). The EdU kit was purchased from RiBo Biological (Guangzhou, China). The protein assay kit was purchased from Bio-Rad (Hercules, CA). All other chemicals used were of analytic grade.

For cellular experiments, #2714 was dissolved in DMSO stock solution for all in vitro assays and stored in the dark at 4 °C. The initially concentration was 40 mM, which was diluted in the cell culture medium with the final concentration of DMSO no more than 0.05% (V/V). For animal experiments in vivo, #2714 was dissolved in ultrapure water and Cremophor EL/ethanol (50:50; Sigma Cremophor EL, 95% ethyl alcohol) mixture, and administrated via i.p. injection to the experimental animals at a dose of 10 ml/kg body weight/day.

### Virtual target identification by IFPTarget and ID-score fingerprinting analyses

Target identification of small molecular agent is an important and challenging work for drug discovery and chemical biology. To identify the possible potential targets of #2714, we used a new virtual target-customized identification method that enables substantially improved binding pose prediction named as IFPTarget, which uses an IFP method based on protein–ligand IFP analyses for target-specific interaction analyses and a comprehensive index (Cvalue) for the target ranking test set by CADD^26–28^. In brief, we applied to screen against our established virtual target library that covering 11,863 protein structures contains 2842 unique targets by IFPTarget and ID-score fingerprinting analyses methods to retrieve known targets within the top-ranked list and identified new possible potential targets for #2714.

IFPTarget prediction led to the identification of 6875 protein structures contains 2842 unique targets for #2714 as validated, and top-ranked list 100 such as Odorant binding protein, antennal, ProCaspase-6, soluble epoxide hydrolase, peroxisome proliferator-activated receptor γ, Ser/thr-transferase B-raf (V600E), MAP4K4, caspase-7 and CDK2. We profiled enzymatic kinase inhibition activity and found that#2714 could inhibit the activities of CDK2 and MAP4K4. From the results of the present study, we speculate that cell cycle arrest- and apoptosis-related protein might be the new potential primary target for #2714.

### Cell lines and cell culture

LL/2 cells, 4T1 (mouse mammary carcinoma cells), NIH-3T3 (mouse fibroblast cell), and HK-2 (human proximal tubular epithelial cells) were obtained from American Type Culture Collection (ATCC, Manassas, VA) and the Chinese Academy of Sciences Typical Culture Preservation Committee Cell Bank (Shanghai, China), cultured in Roswell Park Memorial Institute (RPMI) 1640 medium or Dulbecco’s modified Eagle’s medium (DMEM) plus 10% fetal bovine serum (FBS), 100 units/mL penicillin, and 100 units/mL streptomycin. All cells were cultured in a cell culture chamber with a humidified atmosphere at 37 °C and 5% CO_2_.

### Cell viability assay

Cells (3 × 10^3^) were seeded in 96-well plates and cultured overnight with various designated concentrations of #2714. Cells treated with only cell culture medium and solvent served as controls. After the cells were treated for 48 h, 100 μL culture medium containing 10 μL CCK-8 solution was added to each well and incubated for 2–4 h at 37 °C in the dark. Furthermore, the inhibitory effects of #2714 on LL/2 cells were measured via CCK-8 assay after treatment for 24, 48, and 72 h with various concentrations of #2714. The optical density (OD) was measured at 450 nm using a multiscan spectrum instrument (Thermo Lab Systems, USA). The IC_50_ values of #2714 were calculated using IC_50_ software. The experiment was done in triplicate.

### EdU–DNA incorporation assay

The EdU–DNA incorporation detection assay kit (RiBo Bio, Guangzhou) was used to investigate the labeling of proliferating LL/2 cells. EdU incorporates into chromosomal and replicate DNA during the DNA synthesis S-phase and stains red. The assay was performed in accordance with the manufacturer’s instructions. In brief, LL/2 cells (3 × 10^3^ per well) were cultured into 96-well plates overnight, after which #2714 was diluted into different final concentrations (5, 10, and 20 μM) with RPMI-1640 culture medium, added to the plates, and incubated for 48 h. Thereafter, EdU solutions were added to the plates and incubated for 2–3 h in the dark at 37 °C to enable DNA synthesis and EdU incorporation into the DNA. The plates were then washed and observed using an inverted fluorescent microscope (Carl Zeiss, Germany) with a camera system (Olympus, Tokyo, Japan). The experiment was done in triplicate.

### Cell cycle and apoptosis analysis by FCM

LL/2 cells were stained with PI and analyzed via FCM to determine the effects of #2714 on the cell cycle. Cell culture and drug treatment were done over 48 h, as described above. After treatment with 1 ml hypotonic fluorochrome solution containing 50 ng/ml PI dissolved in 0.1% sodium citrate plus 0.1% Triton X-100, cell cycle stages and apoptosis cells were immediately analyzed via FCM (ESP Elite, Beckman-Coulter, Miami, FL, USA).

Furthermore, to determine if alterations in mitochondrial membrane potential (ΔΨm) were involved in #2714-induced apoptosis and cell cycle arrest, cellular ΔΨms were detected via FCM using the Δψm-sensitive dye Rho 123^31^. Briefly, cells were plated onto six-well plates at a density of 1 × 10^5^ cells per well and allowed to attach for 24 h. Subsequently, #2714 treatments was performed for 48 h, as described above. The cells were then exposed to Rho 123 (5 μg/mL) and kept in the dark in the culture incubator at 37 °C. After 30 min, the cells were washed with phosphate-buffered saline (PBS) and collected with 0.1% sodium citrate plus 0.1% Triton X-100. The changes in Δψm were analyzed via FCM, which detected the fluorescence emitted from the indicator Rho 123 with a single beam at 488 nm excitation and 530 nm emission.

### Western blotting analysis and extraction of cell proteins

Western blotting analysis was modified to determine the possible mechanism of #2714^32,33^. In brief, LL/2 cells were exposed to #2714 for 24 h, as described above. Subsequently, PBS and radioimmunoprecipitation assay (RIPA) buffer were used to wash and lyse the cells. The lysates were then centrifuged at 12,000 × *g* for 30 min at 4 °C. After quantitative analysis via Coomassie Brilliant Blue G-250 protein assay kit (Bio-Rad, USA) and denaturation in boiling water for 5 min, the samples with load buffer were subjected to sodium dodecyl sulfate polyacrylamide gel electrophoresis (SDS-PAGE), transferred to polyvinylidene fluoride (PVDF) membranes (Millipore, MA), and successively incubated with the corresponding primary and second antibodies according to the manufacturer’s recommendations. β-Actin was used as a loading control. Protein bands on the PVDF membranes were observed using a commercially available enhanced chemiluminescence kit (Amersham Biosciences Corp., Piscataway, NJ) and were developed on Aermen X-ray films (Aermen X-ray film Corp, Sichuan).

### Subacute toxicity study of #2714 in C57BL/6 mice

To evaluate the safety profile of #2714 in vivo, C57BL/6 mice (18–22 g), obtained from Beijing laboratory animal cancer (Beijing, China) were used. All animals were raised with free access to the food and water and were housed in controlled environmental conditions in a specific-pathogen-free (SPF) facility with consistent room temperature (25 °C) and humidity. In the present study, the male and female mice (*n* = 10, respectively) were i.p. administered #2714 (25–100 mg/kg/day) for 14 days or a single dose of 1000 mg/kg for the acute toxicity test. Clinical symptoms of all experimental animals were observed once daily for 2 weeks. After, the animals were killed and the main organ tissues and blood were collected via necropsy on the acute toxicity test day. The changes in blood serum biochemistry and hematological analysis were detected using a Hitachi 7200 Blood Chemistry Analyzer (Hitachi, Japan) and a Nihon Kohden MEK-5216K Automatic Hematology Analyzer (Nihon, Japan). The histopathological analyses were carried out using hematoxylin and eosin staining after the heart, liver, spleen, lungs, and kidney were fixed in paraformaldehyde and embedded in paraffin.

All animal experiments in the present study were conducted in full compliance with the institutional animal care and use committee of our university, and carried out in accordance with the National Institutes of Health Guide for the Care and Use of Laboratory Animals.

### In vivo analysis of the #2714 pharmacodynamic profile

In the present study, 6- to 8-week-old female C57BL/6 mice obtained from Beijing animal cancer (Beijing, China) were housed under controlled environmental conditions in a SPF facility, consistent room temperature (25 °C) and humidity, for evaluation of the pharmacodynamic profile of #2714 in vivo. Briefly, 1 × 10^6^ LL/2 tumor cells in 100 µL serum-free RPMI-1640 medium were implanted subcutaneously into the right flank of female C57BL/6 mice^34,35^. After the tumors were allowed to grow freely for 10 days and tumor volumes reached approximately 100 mm^3^, the tumor-bearing mice were randomly sorted into three groups (10 animals per group), and administered #2714 at doses of 25, 50, 100 mg/kg, or vehicle via i.p. injection once daily for 4 weeks, respectively. Tumor volume was measured using Vernier calipers every 3 days, and calculated according to the following formula: tumor volume (mm^3^) = 0.52 × length × width^2^. Body weights were measured every 3 days and clinic symptoms of all the experimental animals were observed once daily.

For immunofluorescent (IF) and immunohistochemical (IHC) analyses, all the experimental animals were killed at the end of the experiment, after having received #2714 for 2 weeks. The tissue sections of LL/2 cell models were collected, fixed with dry ice or 4% paraformaldehyde, and embedded in paraffin, as described previously. Paraffin tumor sections were stained with anti-CD105 antibody using IHC staining to detect cell angiogenesis in tumor tissue, respectively. Frozen tumor sections were stained with anti-CD31 antibody using IF staining to detect the density of angiogenesis in tumor tissue. Images were taken with an inverted fluorescence microscope with a Leica microscope digital camera (Lecia, Germany).

### TUNEL detection in vivo

To examine the apoptosis induction effect of #2714 on tumor cells in vivo, the TUNEL assay was used to analyze the apoptotic cells in the tumor tissue. In brief, an apoptotic cell detection kit (Promega) was used to stain the paraffin tumor sections collected as described above, according to the manufacturer’s recommendations^12^. Fluorescence pictures were taken using an inverted fluorescence microscope with a camera system (Olympus, Tokyo, Japan).

### Statistical analysis

Data are expressed as mean ± SD/SEM and were analyzed by SPSS 13.0 software (Chicago, IL, USA). Student’s two-tailed *t*-test was used to analysis the differences between different groups. Kaplan–Meier curves were applied to analyze survival tests with log-rank statistic. The *p* < 0.05 was considered to be statistically significant.
